# Kidney Carcinoma Ovarian Metastasis: Review of the Literature

**DOI:** 10.7759/cureus.3620

**Published:** 2018-11-21

**Authors:** Nektarios Koufopoulos, Despoina Nasi, Foteini Antoniadou, Stefania Kokkali, Stamatios Theocharis

**Affiliations:** 1 Department of Pathology, “Saint Savvas” Cancer Hospital, Athens, GRC; 2 Department of Oncology, "Saint Savvas” Cancer Hospital, Athens, GRC; 3 Department of Oncology, “Saint Savvas” Cancer Hospital, Athens, GRC; 4 Department of Pathology, National and Kapodistrian University of Athens, Athens, GRC

**Keywords:** clear cell renal cell carcinoma, papillary renal cell carcinoma, chromophobe renal cell carcinoma, collecting duct carcinoma, metastasis, ovary

## Abstract

Ovarian metastasis is common with secondary tumors representing up to 15% of ovarian neoplasms. The malignancies most commonly involving the ovaries are carcinomas of the stomach, colon, breast, endocervix, endometrium, and lymphoma. Secondary ovarian involvement by kidney carcinoma occurs very rarely and is usually associated with widespread dissemination.

We conducted a review of kidney carcinoma with ovarian metastasis in the literature using the keywords clear cell renal cell carcinoma, papillary renal cell carcinoma, chromophobe renal cell carcinoma collecting duct carcinoma, and ovarian metastasis on Google Scholar and PubMed indices in April 2018, including a case diagnosed in our department. To date, 30 articles presenting 41 cases of kidney carcinoma with ovarian metastasis are reported in the literature. All reviewed cases were analyzed for diagnosis, surgical and systemic therapy, and outcome.

Diagnosis may sometimes be challenging, requiring appropriate immunohistochemical markers in difficult cases. A combination of surgery and adjuvant therapy offers significant benefit in disease control or palliation of symptoms. Due to inconsistency in the reported data, further studies are needed to make safe conclusions regarding survival.

## Introduction and background

Metastasis to the ovaries is common, and secondary tumors represent 7% to 15% of the ovarian neoplasms [1]. The most common malignancies responsible for the secondary involvement of the ovaries include stomach, colon, breast, endocervix, endometrium, as well as lymphoma [2]. In many cases, a known history of a primary neoplasm exists, but the ovarian mass is rarely the initial lesion [3].

Among the different types of kidney carcinomas (KC), clear cell renal cell carcinoma (ccRCC) is the most common histotype. It usually metastasizes to the lungs, lymph nodes, bones, brain, liver [2], and, very rarely, the ovary. According to our knowledge, fewer than 40 cases have been published in the English literature to date.

Papillary renal cell carcinoma (PRCC) and chromophobe renal cell carcinoma (ChRCC) account for 10% and 5% of the KC cases, respectively. They display an indolent behavior, remaining confined to the kidney [4], while collecting duct carcinoma (CDC) [5] and unclassified renal cell carcinoma (RCCU) [6] are rare tumors with aggressive clinical behavior.

Using the keywords clear cell renal cell carcinoma, papillary renal cell carcinoma, chromophobe renal cell carcinoma collecting duct carcinoma, and ovarian metastasis, we reviewed reports of KC with ovarian metastasis via Google Scholar and PubMed indices in April 2018, including a case diagnosed in our department. Herein, we have reviewed the clinicopathological features, treatment, and outcome of the 41 KC cases with ovarian metastasis yielded by our search.

## Review

KC metastasis to the ovary is a rare event. This can be explained by its male predominance with the male-to-female ratio being 2:1 [7], low incidence of tumor emboli to the ovary, vascular sclerosis of the postmenopausal ovary when KC usually occurs, and the misdiagnoses of some metastatic tumors as primary ovarian neoplasms [8-9].

In the literature, several KC cases were not completely analyzed, with important data missing such as the type of surgical operation, adjuvant therapy, and sizes of the primary and metastatic tumors. The clinicopathological features of the cases, 36 ccRCC, two PRCC, one CDC, one ChRCC, and one RCCU, are presented in Table 1. Patients’ age ranged from 17 to 80 years (mean age: 53 years). The right kidney was involved in 20 and the left in 19 patients. Ovarian metastasis was ipsilateral in 13, contralateral in 15, and bilateral in 11 cases, including our case. According to the available data, primary tumor size ranged from 50 mm to 165 mm (mean value: 86 mm), whereas the size of the secondary tumor ranged from microscopic involvement to 180 mm (mean value: 100 mm). In two cases, there was tumor-to-tumor metastasis consisting of two small nodules in a mucinous cystadenoma [10] in the first case and a multilocular cystic ovarian tumor consisting of mixed mucinous cystadenoma and Brenner tumor in the second one [11].

**Table 1:** Kidney carcinoma clinicopathological characteristics NA: not available, SATh: systemic adjuvant therapy, HBSO: hysterectomy and bilateral salpingo-oophorectomy, BSO: bilateral salpingo-oophorectomy, RSO: right salpingo-oophorectomy, LSO: left salpingo-oophorectomy, LO: left oophorectomy, DOD: died of disease, AWD: alive with disease, ANED: alive no evidence of disease, ccRCC: clear cell renal cell carcinoma, ChRRC: chromophobe renal cell carcinoma, CDC: collecting duct carcinoma, PRCC: papillary renal cell carcinoma, RCCU: renal cell carcinoma unclassified. *Four patients in the Liang et al. series received chemotherapy. No more details were provided.

Year	Age	Type	Kidney	Ovary	Surgery	S.A.Th.	Outcome(mo)	Author
1949	57	ccRCC	Left	Left	NA	No	DOD (18)	Martzlof et al. [12]
1957	64	ccRCC	Right	Bilateral	BSO	No	DOD (15)	Hobbs et al. [13]
1981	68	ccRCC	Right	Left	LO	No	ANED (25)	Stefani et al. [14]
1983	52	ccRCC	Left	Left	NA	No	Unknown	Buller et al. [15]
1992	48	ccRCC	Right	Left	LSO	No	AWD (96)	Young et al. [16]
1992	62	ccRCC	Left	Right	HBSO	No	AWD (6)	Young et al. [16]
1992	48	ccRCC	Left	Left	LSO	No	NA	Young et al. [16]
1992	28	ccRCC	Right	Left	NA	NA	NA	Liu et al. [17]
1993	40	ccRCC	Left	Bilateral	HBSO	No	AWD (55)	Spencer et al. [18]
1994	46	ccRCC	Left	Bilateral	HBSO	Yes	ANED (36)	Adachi et al. [19]
1996	54	ccRCC	Right	Left	NA	No	NA	Fields et al. [8]
1998	80	ccRCC	Right	Bilateral	HBSO	No	ANED (24)	Vara et al. [20]
2003	48	ccRCC	Left	Bilateral	HBSO	Yes	AWD (3)	Hammock et al. [21]
2003	50	ccRCC	Right	Right	HBSO	No	ANED (6)	Insabato et al. [10]
2003	49	ccRCC	Right	NA	NA	No	DOD (6)	Insabato et al. [10]
2003	17	ccRCC	Left	Left	NA	No	ANED (24)	Insabato et al. [10]
2004	61	ccRCC	Left	Bilateral	BSO	Yes	AWD (24)	Valappil et al. [22]
2004	79	CDC	Left	Left	No	No	DOD (<1)	Kassouf et al. [23]
2006	52	ccRCC	Left	Right	BSO	Yes	DOD (10)	Kato et al. [24]
2007	73	PRCC	Right	Left	LSO	NA	NA	Stolnicu et al. [25]
2009	56	ccRCC	Right	Bilateral	HBSO	No	ANED (19)	Albrizio et al. [26]
2009	45	ccRCC	Right	Left	LO	Yes	AWD (48)	Anagnostou et al. [27]
2009	54	ccRCC	Left	Left	HBSO	No	DOD (9)	Toquero et al. [7]
2010	54	ccRCC	NA	Bilateral	HBSO	Yes	AWD (48)	Guney et al. [28]
2011	63	ccRCC	Right	ΝΑ	HBSO	No	AWD (132)	Decoene et al. [29]
2012	45	ccRCC	Left	Right	HBSO	Yes	NA	Udoji et al. [30]
2012	71	ccRCC	Right	Right	RSO	No	NA	Ibrahim et al. [11]
2014	61	ccRCC	Right	Bilateral	HBSO	Yes	ANED (12)	Bauerová et al. [31]
2015	55	ccRCC	Left	Left	HBSO	No	ANED (14)	Dolanbay et al. [32]
2015	51	ccRCC	Left	Right	HBSO	No	ANED (2)	Kostrzewa et al. [9]
2015	48	ccRCC	Right	Right	HBSO	No	NA	Bohara et al. [33]
2016	60	ccRCC	Right	Right	NA	*	AWD (40)	Liang et al. [34]
2016	48	ccRCC	Left	Right	NA	*	AWD (57)	Liang et al. [34]
2016	45	ccRCC	Right	Left	NA	*	DOD (48)	Liang et al. [34]
2016	43	ccRCC	Right	Right	NA	*	DOD (109)	Liang et al. [34]
2016	52	ccRCC	Left	Right	NA	*	DOD (132)	Liang et al. [34]
2016	52	ccRCC	Right	Left	NA	*	DOD (204)	Liang et al. [34]
2016	NA	ChRRC	xxx	NA	NA	*	NA	Liang et al. [34]
2016	37	RCCU	Left	Bilateral	NA	*	AWD (22)	Liang et al. [34]
2017	45	PRCC	Left	Left	HBSO	No	ANED (3)	Bashkar et al. [35]
2017	46	ccRCC	Right	Bilateral	HBSO	Yes	DOD (7)	Koufopoulos et al. [36]

Regarding the presenting symptoms, 11 patients were asymptomatic with metastasis found on scheduled postoperative imaging. Seven patients presented with an abdominal mass [7,8,10,16,22,32], four with weight loss [7,11,13,34], three with vaginal bleeding [10,12,19], two with fever [14,20], two with irregular menses [18,21], two with an abdominal distension [11,26], two with ascites [8,15], abdominal pain [20,36], weight gain [15], fatigue [13], weakness [14], shortness of breath [8,36], pelvic pain [26], generalized malaise and decreased energy [30], flank pain [34], bone fracture [24], and thyroid metastasis [16]. In 26 patients, ovarian metastases were detected three months to 21 years after kidney involvement. In six cases, the primary cancer site and the metastasis were discovered concomitantly, while in four cases, the metastasis was detected first. In one case, ovarian metastasis was detected eight years before the primary tumor [12]. Fuhrman grade was mentioned in 11 cases, including our case, four of them being grade 2, six grade 3, and a single case grade 4 with sarcomatoid features [7,24,29,30,33-34].

Clinical features such as cyst formation, size, and bilaterality did not help in differentiating primary from metastatic ovarian tumors [37]. Histologically, the main differential diagnosis of metastatic ccRCC to the ovary was between ovarian clear cell carcinoma (OCCC), steroid cell tumor, dysgerminoma, and clear cell variant of struma ovarii [10,21]. In cases of simultaneous presentation of kidney and ovarian tumor, ovarian metastasis to kidney and independent primaries have to be considered. There are three cases with independent primaries [31] and four ovarian carcinomas metastatic to the kidney [9] in the literature. Attention to histological differences between ccRCC and its ovarian mimics paired with clinical history were considered sufficient on several occasions.

**Figure 1:**
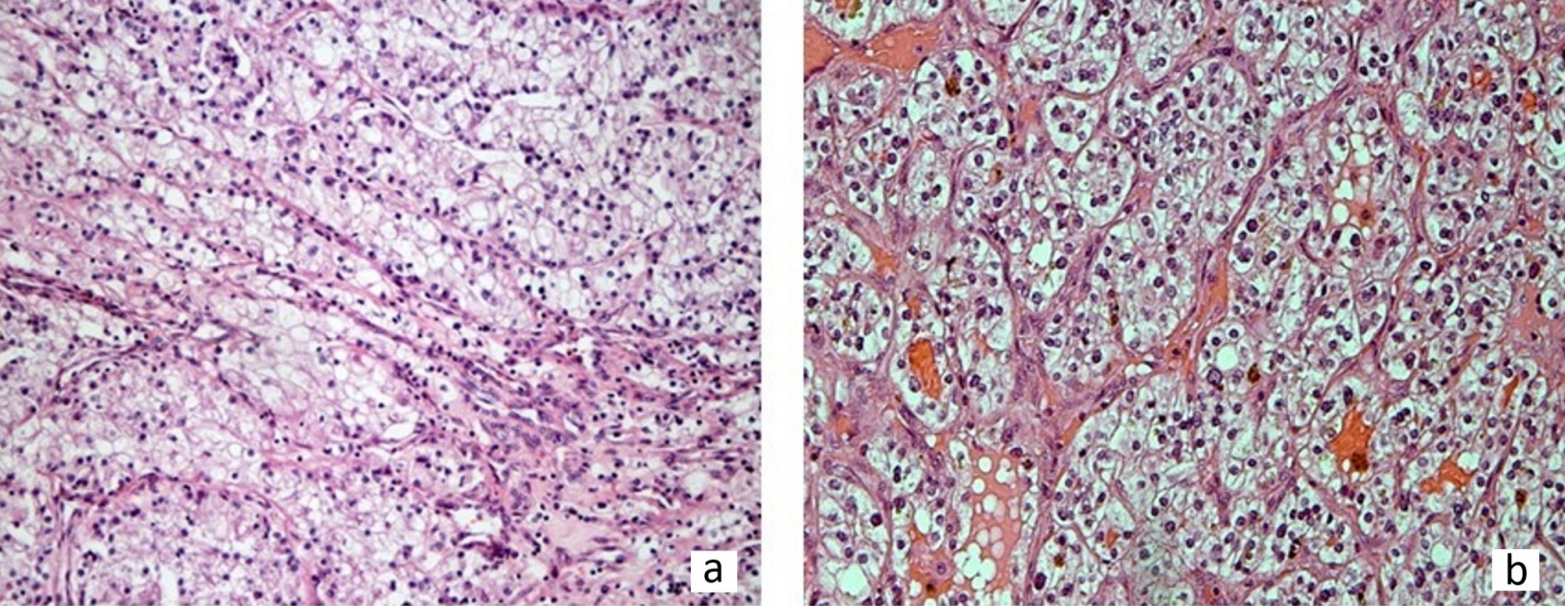
(a) ccRCC tumor cells with moderately atypical nuclei lacking significant pleomorphism (H&E x 100). (b) On higher power magnification, a prominent, thin-walled vascular network is characteristic of ccRCC (H&E x 200). ccRCC: clear cell renal cell carcinoma, H&E: hematoxylin and eosin

Microscopically, both ccRCC and OCCC may have cystic, solid, tubular, and papillary areas. Tumor cells may display clear or eosinophilic cytoplasm and prominent nucleoli. In ccRCC, tumor cells usually lack significant pleomorphism (Figure 1a) and are characteristically associated with a prominent vascular network (Figure 1b) [18,22]. In OCCC, tubular or glandular formations are lined by hobnail cells at least focally in almost 90% of cases [20]. Tubules and cysts in OCCC may be filled with extracellular mucin [18,20], a feature not present in ccRCC.

Steroid cell tumors not otherwise specified are composed of lipid-rich tumor cells with clear intracytoplasmic vacuoles that are arranged in solid sheets, thin cords, or columns lacking the tubular differentiation of ccRCC which often contain intraluminal blood or colloid-like material [10,21].

Dysgerminomas display a diffuse, trabecular, insular, or cordlike pattern. Tumor cells have large uniform round nuclei and clear cytoplasm. The stroma is filled with numerous mature lymphocytes [21]. The clear cell variant of struma ovarii lacks the characteristic vascular pattern of ccRCC [10].

**Figure 2:**
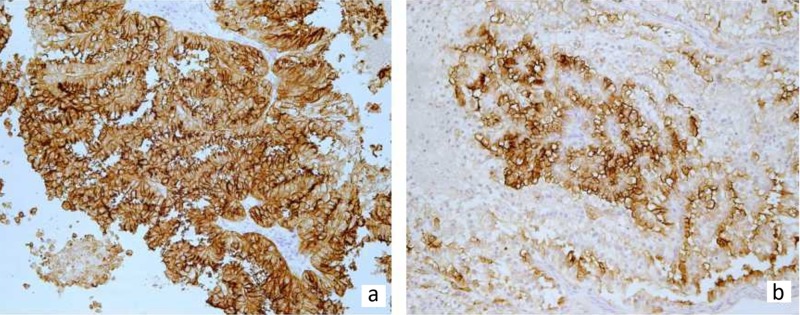
(a) CD-10 is usually diffusely positive in ccRCC cells (CD-10 x 200). (b) Tumor cells showing positive staining for RCCma (RCCma x 200) ccRCC: clear cell renal cell carcinoma, RCCma: renal cell carcinoma marker

In difficult cases when ovarian metastasis is the first presentation of the disease, immunohistochemistry can provide diagnostic solutions, and such an approach was followed in 11 of 35 published cases [10-11,21,26-27,31-34,36]. Among the most useful immunohistochemical markers are cluster of differentiation (CD)-10 (Figure 2a) and RCCma (Figure 2b). The immunohistochemical profile of ccRCC and its ovarian histological mimics is reported in Table 2.

**Table 2:** Immunophenotypic profile of clear cell tumors of the ovary CK7: cytokeratin 7, EMA: epithelial membrane antigen, OCCC: ovarian clear cell carcinoma, ccRCC: clear renal cell carcinoma, PLAP: placental alkaline phosphatase, RCCma: renal cell carcinoma marker

	AE1/3	CK7	EMA	CD-10	RCCma	Inhibin	PLAP	CD-117
OCCC	+	+	+	-	-	-	-	-
Metastatic ccRCC	+	-	+	+	+	-	-	-
Steroid cell tumor	-	-	-	-	-	+	-	-
Dysgerminoma	-	-	-	-	-	-	+	+

In the literature, all 41 patients were treated surgically, 14 with hysterectomy and bilateral salpingo-oophorectomy (HBSO), four with bilateral salpingo-oophorectomy, two with left salpingo-oophorectomy (LSO), one with right salpingo-oophorectomy, and two with left oophorectomy. In 12 cases, no information concerning the type of surgical procedure was provided. Surgical treatment seems to offer a significant benefit in disease-free and overall survival (OS) in metastatic ccRCC. However, in the contemporary era of targeted therapy, cytoreductive nephrectomy or metastasectomy lacks proof of survival benefit with randomized trials [38]. Furthermore, complete resection may be predictive of prolonged OS, along with the number of metastatic lesions (>1 lesion), synchronous or asynchronous disease (>1 year from nephrectomy to metastatic disease), site of metastases (pulmonary vs. extrapulmonary), and age (younger vs older than 60 years) [39-40].

Adjuvant therapy was reported in 12 patients in the literature. Eight patients received systemic therapy with interferon-a [19,24], interleukin-2 [22], a combination of interferon-a and sunitinib [27], sunitinib [28], and vinblastine [31]. A patient started treatment with interleukin-2 and continued with a high dose of sunitinib after recurrence. Due to a lack of response, the regimen was altered to everolimus, resulting in no disease progression [30]. In one case, the patient received three cycles of chemotherapy with no further specification [21]. Liang et al. mention four patients receiving chemotherapy without providing more details [34]. Systemic adjuvant therapy was used in our case [36] consisting of the administration of sunitinib initially, later changed to sorafenib, which was stopped two months later due to intolerance and toxicity. Subsequently, pazopanib was administered showing a poor response, followed by nivolumab until the patient succumbed to the disease. Radiotherapy was mentioned in four cases, two of them as palliative therapy for bone [7,29] and brain metastases [9] and in one case for parotid gland, thyroid, and brain metastases [18]. Follow-up was available in 28 patients with the longest period lasting 204 months. Ovarian metastasis is usually part of generalized disease with several patients also having metastases to other organs prior to, concurrently, or after ovarian involvement. The most frequent metastatic sites were, by descending order, bone in five cases [7,10,17,24,30], lung in four cases [7,13,24], abdomen in four cases [10,13,22,30], adrenal glands in three cases [27,34], skin in two cases [18,22], muscle in two cases [20,34], thyroid in two cases [9,16], brain in two cases [9,18]; and one metastasis to each of the following organs: parotid [18], liver [34], gallbladder [29], vagina [12], and cervical, pelvic, and paraaortic lymph nodes [7,16,22]. In 12 cases, the ovary was the sole metastatic lesion described. Ten patients died of disease, nine patients were alive with no evidence of disease (ANED), and nine were alive with metastatic disease (AWD) in a period ranging from two months to 17 years (mean: 44 months).

Because there is no consistency in the data reported by different authors, no safe conclusions can be made regarding survival.

Two cases of PRCC ovarian metastasis were found in the literature. Patients’ age ranged from 45 to 73 years (mean age: 59 years). The right and left kidneys were involved in one case each. Ovarian metastasis was ipsilateral in one case [35] and contralateral in the second [25]. The primary tumor size ranged from 85 to 106 mm (mean value: 95.5 mm), whereas the size of the metastatic lesion ranged from 60 to 117 mm (mean value: 88.5 mm). Patients presented with abdominal pain, constipation [25], and abdominal mass [35]. Ovarian metastasis was detected 36 months after kidney involvement [25] and concomitantly [35]. Fuhrman grade is mentioned in one case (grade 3). Simultaneous metastatic involvement was present in both cases in the para-aortic lymph nodes [35] and anterior parietal peritoneum [25]. Patients were treated with HBSO [35] and LSO [25]. No other clinical data concerning treatment or follow-up are referred [25] in one case, whereas the other patient did not receive adjuvant therapy and was ANED three months after surgery [35].

Histologically, PRCC may simulate papillary tumors from other locations, including the ovary such as in OCCC and ovarian serous carcinoma [25,34]. In difficult cases, immunohistochemical staining may provide some help. Typical high-grade serous carcinoma is positive for Wilms' tumor 1 (WT-1), and therefore negative staining for WT-1 favors metastatic carcinoma [34].

A single case of metastatic ChRCC to the ovary is described in a case series [34]. A patient with a history of ChRCC presented with bilateral ovarian metastases found on scheduled postoperative imaging. The tumor of the right ovary measured 150 mm, while the one of the left measured 55 mm. No other clinical data were available. ChRCC ovarian metastasis can mimic sex cord-stromal tumors. ChRCC will stain positive for CD-117 and negative for estrogen receptor, calretinin, and inhibin [34].

A single case of metastatic CDC to the ovary was found in the English literature. A 79-year-old patient presented with symptoms of pyelonephritis and hypercalcemia. A radical nephrectomy was performed. High-grade malignant cells were found on frozen sections of the lymph nodes and psoas mass. There was also a large mass in the pelvis close to the primary tumor. Pathology showed a CDC. The patient had postoperative complications, dying 27 days after surgery. Metastatic disease to the left ovary was found at autopsy [23]. CDC differentiation from primary ovarian carcinomas may be difficult due to overlapping histological features such as tubulopapillary architecture and hobnailing. Immunohistochemistry may provide little help with cytokeratin (CK)-903, CK-19, Ulex europaeus agglutinin lectin, and vimentin being positive in CDC [5].

Histologically, it had a papillary architecture mimicking ovarian serous carcinoma and was immunopositive for paired box gene 8 (PAX8), vimentin, P504S, P53, and CK20 (focal) and negative for CK7, WT-1, high molecular weight cytokeratin and p63, CK5/6, CD10, and estrogen receptors, supporting the diagnosis of RCCU. There was also involvement of the omentum, peritoneum, and abdominal wall. The patient was AWD 22 months after surgery.

## Conclusions

Ovarian metastasis of KC occurs very rarely. Precise diagnosis may occasionally be challenging, but it is essential for the appropriate management. Immunohistochemistry will assist in most cases. Surgery seems to be an effective means to treat KC metastasis, offering a significant benefit in a disease-free and cancer-specific OS in retrospective studies so far. Adjuvant therapy offers significant benefit in disease control or palliation of symptoms.
